# Exploring the bioavailability and bioactivity of thiamine versus allithiamine: studies in *Drosophila melanogaster* and mice

**DOI:** 10.3389/fnut.2026.1812854

**Published:** 2026-06-24

**Authors:** Sandra Nevermann, Hannah Weiß, Alexandra Fischer, Kai Lüersen, Keita Chikamoto, Daisuke Nakata, Yoshiyuki Ishida, Takahiro Furune, Keiji Terao, Gerald Rimbach

**Affiliations:** 1 Department of Food Science, Institute of Human Nutrition and Food Science, Kiel University, Kiel, Germany; 2 CycloChem Bio Co., Ltd., Kobe, Japan

**Keywords:** allithiamine, *Drosophila melanogaster*, HPLC, mice, thiamine, vitamin B_1_

## Abstract

**Objective:**

Thiamine (vitamin B_1_) is an essential micronutrient involved in carbohydrate metabolism and mitochondrial function. Allithiamine, a lipid-soluble thiamine derivative, has been proposed to exhibit thiamine bioactivity.

**Methods:**

Here, we conducted complementary studies in *Drosophila melanogaster* and laboratory mice to compare the bioavailability and bioactivity of thiamine and allithiamine. First *Drosophila* was established as a whole-organism model to assess thiamine activity. Flies were reared on a chemically defined, thiamine-free medium supplemented with thiamine, allithiamine or benfotiamine as a lipophilic reference compound. Developmental progression was monitored from egg to adult. In a subsequent mouse experiment, growing thiamine-deficient mice were supplemented with either thiamine or allithiamine up to 7 days. Thiamine status was determined by HPLC-based quantification of whole blood thiamine levels, in parallel with assessments of metabolic and enzymatic parameters.

**Results:**

In flies, thiamine deficiency caused developmental arrest at the first-instar larval stage, whereas supplementation with thiamine or allithiamine fully restored normal development. Notably, HPLC analysis revealed slightly higher tissue thiamine levels following allithiamine supplementation. In thiamine-depleted mice, supplementation with either thiamine or allithiamine resulted in similarly elevated whole blood thiamine levels and increased phosphorylated pyruvate dehydrogenase E1 subunit alpha (pPDHA1) to total PDHA1 ratios. Mitochondrial oxidative phosphorylation (OXPHOS) related protein levels remained largely unchanged, except for moderate increases in ATP synthase F1 subunit alpha (ATP5A). Likewise, only minor effects on mRNA levels of thiamine-related genes were observed following supplementation with either vitamer.

**Conclusion:**

Together, these findings provide the first direct *in vivo* comparison of thiamine and allithiamine across two complementary animal models. Thiamine and allithiamine exhibited comparable bioavailability and biological activity under controlled experimental conditions, while revealing subtle differences in early metabolic regulation. These results establish allithiamine as a biologically active thiamine vitamer *in vivo*. They further highlight post-translational regulation as a key mechanism governing metabolic adaptation to thiamine repletion with implications for nutrition research and the evaluation of thiamine vitamers in dietary strategies.

## 1 Introduction

The water-soluble vitamin thiamine (vitamin B_1_) is an essential cofactor in numerous biochemical pathways. It plays a crucial role in carbohydrate metabolism by regulating key enzymes, including pyruvate dehydrogenase, transketolase, and *α*-ketoglutarate dehydrogenase. These enzymes are central to the citrate cycle and the pentose phosphate pathway (1). Major dietary sources of thiamine include meat, fish, grains, legumes, nuts, and fortified breakfast cereals. As thiamine is water-soluble and only stored in limited amounts in the body, regular dietary intake is required (2–4). Thiamine deficiency leads to severe metabolic dysfunction, manifesting as developmental impairments, neurological disorders, and systemic physiological disturbances (5, 6). Both chronic alcohol consumption and diabetes increase the risk of thiamine deficiency (7–9).

Excessive alcohol consumption impairs intestinal thiamine absorption and represents a major risk factor for Wernicke’s encephalopathy (7, 8). In individuals with diabetes, hyperglycemia increases the metabolic demand for thiamine due to its role in glucose metabolism and simultaneously enhances renal thiamine excretion (9, 10). In addition, pharmaceuticals such as metformin have been shown to inhibit intestinal thiamine transporter, further contributing to thiamine deficiency (11). In both contexts, insufficient thiamine status is commonly addressed through supplementation with thiamine or lipophilic thiamine derivatives (9, 12).

**Figure 1:**
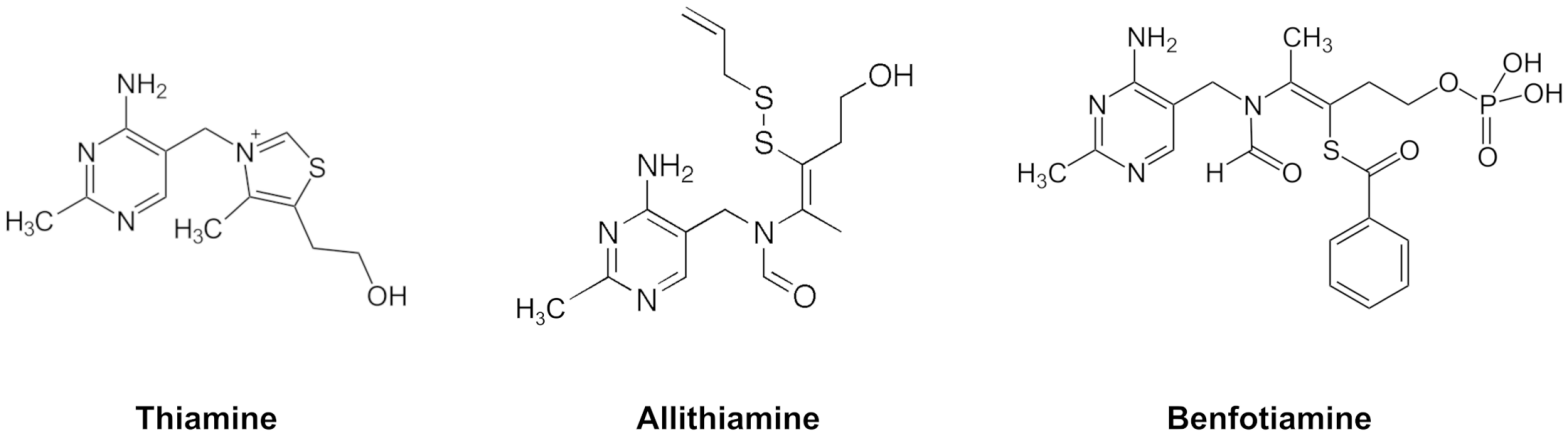
Chemical structures of thiamine, allithiamine and benfotiamine.

Allithiamine (chemical structures shown in Figure 1) is a lipophilic derivative of thiamine formed through the reaction of thiamine with allicin, a reactive organosulfur compound naturally occurring in garlic (*Allium sativum*) and other *Allium* species (13). Mechanical disruption of garlic tissue activates the enzyme alliinase, which converts the sulfur-containing amino acid alliin into allicin. Allicin can then react with the thiazolium ring of thiamine to form thiamine disulfide derivatives, including allithiamine (14). This non-enzymatic modification requires both thiamine and allicin. It is further facilitated by conditions such as tissue disruption and alliinase activity.

Due to its increased lipophilicity and enhanced membrane permeability, allithiamine is hypothesized to exhibit improved intestinal absorption and more efficient distribution to target tissues compared to water-soluble thiamine (15). These properties suggest a potential advantage over conventional thiamine. However, direct comparative *in vivo* studies of thiamine and allithiamine under controlled conditions remain scarce. In particular, comparative data linking vitamer availability to functional development and metabolic outcomes are lacking. To address this gap, complementary model systems are required that enable for both developmental and metabolic assessment.

The fruit fly *Drosophila melanogaster* is a well-established model organism in experimental research due to its well-characterized genetics, short generation time, and high sensitivity to dietary factors. Approximately 60% of *Drosophila* genes have mammalian orthologs, resulting in highly conserved metabolic and signal transduction pathways (16). Importantly, thiamine is an essential micronutrient for *Drosophila melanogaster* (17).

In this study, we introduced *Drosophila* as a whole-organism model to investigate the bioactivity of thiamine and its lipophilic derivative allithiamine *in vivo*. *D. melanogaster* was used to evaluate developmental responses across a range of concentrations. This approach enabled rapid and cost-effective screening of micronutrient bioactivity. Specifically, we assessed the impact of dietary thiamine deprivation and supplementation on larval development, and compared these outcomes to those observed with allithiamine supplementation.

Based on the findings obtained in *Drosophila*, we subsequently conducted a targeted murine study using a single, physiologically relevant concentration of either thiamine or allithiamine in growing mice, a developmental stage characterized by increased thiamine demand. This mouse model enabled the assessment of whole-blood thiamine levels, systemic bioavailability, and metabolic efficacy in a mammalian system.

The sequential use of *Drosophila* and mice thus provided a complementary framework for evaluating the *in vivo* bioactivity of thiamine versus allithiamine from both developmental and metabolic perspectives. We hypothesized that thiamine and allithiamine would exert comparable impacts on developmental outcomes in *D. melanogaster*. We further expected similar whole-blood thiamine levels in mice despite differences in physicochemical properties and pharmacokinetics.

## 2 Materials and methods

### 2.1 *Drosophila melanogaster* experiments

#### 2.1.1 Strain and culture conditions

The *D. melanogaster* strain *w^1118^* (#5905; Bloomington *Drosophila* Stock Center, Indiana University, USA) was used in all experiments. Flies were maintained in climate chambers (Memmert GmbH + Co. KG, Germany) under standardized conditions: 25 °C, 60% relative humidity, and a 12 h light/dark cycle. Until egg collection, flies were maintained on standard culture medium as described by Wagner et al. (18), composed of 5.5% dextrose, 3.0% sucrose (Carl Roth, Germany), 6.0% cornmeal, 2.5% inactive dry yeast, 1.0% agar Type II (Kisker, Germany), 0.1% Tegosept (Genesee Scientific, USA), and 0.3% propionic acid (Carl Roth, Germany).

#### 2.1.2 Experimental diets

*Drosophila* experiments were conducted using a chemically defined, thiamine-free medium. Preparation of the medium based on the FLYaa formulation described by Piper et al. (19) and the HolFast medium reported by Sorge et al. (20) whereby thiamine was omitted from the vitamin stock solution. The medium was used either without a further supplementation (B_1_-free) or with supplementation with thiamine hydrochloride (Carl Roth, Germany) at concentrations ranging from 0.1 to 1.0 μmol/L. For comparative analyses, allithiamine [ChiroBlock, Germany; customized synthetization according to Biro et al. (21)] or benfotiamine (Biosynth Ltd., United Kingdom) were added at a concentration of 0.5 μmol/L. In all supplemented conditions, the volume of the respective compound solution was added in the exchange of water to maintain a constant final volume.

#### 2.1.3 Assessment of developmental parameters

Synchronized eggs collected from grape agar plates (Genesee Scientific, USA) were transferred on the various experimental diets. Developmental progression from egg to adult was monitored daily at the same time. Images were captured using an ocular camera (Dino-Lite, Netherlands) to determine the developmental progress and size of the pupa (DinoCapture 2.0 software, Dino-Lite).

### 2.2 *Mus musculus* experiments

#### 2.2.1 Animals, experimental design and diets

A total of 72 male C57BL/6NRj mice, aged 3 weeks, were purchased from Janvier Labs (Saint-Berthevin, France). Mice were single housed in individually ventilated cages (IVC) with environmental enrichment and under controlled conditions (22 °C–24 °C, 50%–55% relative humidity, 12 h light/dark cycle). Mice had *ad libitum* access to tap water and food. The animal study was performed according to the international and national regulations of animal welfare. The experimental protocol was permitted by the responsible authority (V 242-39,331/2022 (42-5/22); Ministry of Energy, Agriculture, the Environment, Nature and Digitalization, Schleswig–Holstein, Germany).

During 5 days of habituation, all mice received a standard maintenance diet (#V1534-000; ssniff Spezialdiaeten GmbH, Germany), followed by a thiamine-free diet (ssniff GmbH; Table 1) for 1 week. After this period, mice were assigned to one of five groups (*n* = 8 animals per group) based on body weight: a thiamine-free group (B_1_-free) and two supplementation groups receiving either thiamine (B_1_) or allithiamine (AT). The supplementation groups were further divided according to feeding durations of 1 and 7 days with the respective supplemented diet (ssniff GmbH; Table 1), resulting in the B_1_-d1, B_1_-d7, AT-d1, and AT-d7 groups (Table 2). Food intake was determined daily and body weight gain weekdays.

**Table 1:** Composition of the experimental diets used in the mice study.

Ingredients	Thiamine-free diet	Thiamine diet	Allithiamine diet
Corn starch (%)	24.0	24.0	24.0
Sucrose (%)	30.6	30.6	30.6
Maltodextrin (%)	8.0	8.0	8.0
Cellulose powder (%)	6.0	6.0	6.0
Thiamine mononitrate (mg/kg)	—	3.0	—
Allithiamine (mg/kg)	—	—	3.3*
Crude protein (%)	15.8	15.8	15.8
Crude fat (%)	7.0	7.0	7.0
Crude fiber (%)	6.0	6.0	6.0
Crude ash (%)	4.8	4.8	4.8
Energy (MJ/kg)	16.3	16.3	16.3

*Equimolar amount of thiamine mononitrate.

**Table 2:** Experimental groups of the mice feeding study.

Group	*n*	Diet	Supplementation duration
B_1_-free	8	Thiamine-free diet	—
B_1_-d1	8	Thiamine diet	1 day
B_1_-d7	8	Thiamine diet	7 days
AT-d1	8	Allithiamine diet	1 day
AT-d7	8	Allithiamine diet	7 days

At the end of the experimental periods, mice were fasted for 4 hours and euthanized in their home cages by CO_2_ inhalation using a gradual chamber volume displacement rate of 39% per minute. Blood was subsequently collected by cardiac puncture into heparin-containing tubes. An aliquot of whole blood was snap-frozen in liquid nitrogen for thiamine quantification, while the remaining blood was centrifuged to obtain plasma. Whole blood and plasma samples were stored at −80 °C until analysis. Tissue samples were either snap-frozen in liquid nitrogen and stored at −80 °C or preserved in RNAlater (#R0901, Sigma-Aldrich) and stored at −20 °C.

#### 2.2.2 RNA isolation and quantitative reverse transcription polymerase chain reaction

Total RNA was isolated from liver tissue and intestinal mucosa using peqGOLD TriFast (VWR International, Germany) following the manufacturer’s protocol. The quality and quantity of the RNA were determined with a NanoDrop 2000 (Thermo Fisher Scientific GmbH, Germany) by measuring the absorbance at 230, 260 and 280 nm. Quantitative Reverse Transcription Polymerase Chain Reaction (qRT-PCR) was carried out with the SensiFAST SYBR No-ROX One-step Kit (Bioline, Germany) using the Rotor-Gene 6000 thermocycler (Corbett Life Science, Australia). Primer sequences (Eurofins Genomics, Germany) used for qRT-PCR are given in Table 3. Relative mRNA levels of target genes were normalized to 18sRNA and *β*-actin gene expression.

**Table 3:** Primer sequences used for qRT-PCR in the mouse study.

Gene	Forward (5′–3′)	Reverse (5′–3′)
18sRNA	GGTAACCCGTTGAACCCCAT	CAACGCAAGCTTATGACCCG
β-actin	GACAGGATGCAGAAGAGATTACT	TGATCCACATCTGCTGGAAGGT
Slc19a2	GTTCCTCACGCCCTACCTTC	GCATGAACCACGTCACAATC
Slc19a3	GTCCTATGGGAACACAAGGCA	ATGAAGCCAAAGCTCCTCCAA
Slc22a1	TCCTGTGTGAACTTGGGCTT	GAGCTTACGGCCAAACCTGT
Slc25a19	TCAGTGTCAGGATTTGTCACCCGT	AGAATGCTCTTGGGCCTTCCTCTT
Tkt	CAGCCAACACAAAGGGCATT	ATGCAGAGTTACACCAGCCC
Tpk1	CCACCCGCCATTGTAATCCA	GGCGAAATCGTGCATCCAAA

#### 2.2.3 Western blot analysis

Protein levels of total pyruvate dehydrogenase E1 subunit alpha (PDHA1) and phosphorylated PDHA1 at Ser 293 (pPDHA1) were analyzed in the cytosolic fraction prepared from fresh liver tissue, while representative subunits of mitochondrial oxidative phosphorylation (OXPHOS) complexes I–V were determined in whole cell lysates from frozen liver tissue as previously described (22, 23). Protein concentration was measured with the Pierce bicinchoninic acid (BCA) Protein Assay Kit (Thermo Fisher Scientific, Germany) according to the manufacturer’s instructions.

For Western blot analysis, 30 μg protein per sample were mixed with loading buffer, denatured at 95 °C for 5 min (PDHA1 and pPDHA1) or at 37 °C for 20 min (OXPHOS complexes) and separated on a TGX Stain-Free Precast gradient gel (Bio-Rad Laboratories GmbH, Germany). Proteins were transferred onto a PVDF membrane, which was blocked with 3% bovine serum albumin in TBS containing 0.05% Tween-20 and incubated overnight with a primary antibody (phosphor-PDHA1-S293: 1:10000, AP1022, ABclonal Germany GmbH, Germany; pyruvate dehydrogenase E1: 1:1000, C51G1, Cell Signaling, Netherlands; OXPHOS antibody cocktail: 1:250, ab110413, Abcam Limited, United Kingdom), followed by a secondary antibody (anti-rabbit, 1:4000, 1705046, Bio-Rad; anti-mouse, 1:4000, 1705047, Bio-Rad). For detection of total and phosphorylated PDHA1, membranes were sequentially probed following stripping procedures, allowing analysis of both targets on the same membrane. The protein bands were visualized with ECL reagent (Thermo Fisher Scientific, Germany) in a ChemiDoc Go system (Bio-Rad). Band intensities of target proteins were quantified and normalized to total protein load per lane, measured as membrane fluorescence, with Image Lab software (Version 6.1.0, Bio-Rad).

### 2.3 Quantification of thiamine and its mono- and pyrophosphate esters in *Drosophila* larvae and murine whole blood

Quantification of thiamine (B_1_), thiamine monophosphate (TMP) and thiamine pyrophosphate (TPP) levels in samples from wandering third-instar larvae (L3) of *D. melanogaster* and whole blood samples from mice were performed by HPLC with fluorescence detection, based on the method of Whitfield et al. (24) with modifications, using a Jasco HPLC system (2000 series; Jasco Deutschland GmbH, Germany).

*Drosophila* larvae (five L3 pooled per sample) were homogenized in 100 μL of 10% trichloroacetic acid (TCA) using a TissueLyserII (Qiagen, Germany). Frozen heparinized whole-blood samples from mice were thawed on ice in the dark. Samples were mixed 1:1 with 10% TCA containing amprolium (5μM; TCA/Amp), which served as an internal standard as reported by Zhang et al. (25). Blood and tissue samples were then mixed vigorous for 1 min, incubated in the dark and on ice for 15 min, and centrifuged at 13,000×*g* for 6 min at 4 °C. Subsequently, 80 μL of the supernatant were transferred to a new tube and kept on ice until derivatization.

For derivatization, samples were equilibrated to room temperature and mixed with 20 μL methanol to enhance the fluorescence signal, followed by the addition of 50 μL derivatization reagent (0.04% potassium ferricyanide in 15% NaOH) to convert thiamine and its phosphate esters into fluorescent thiochromes. After incubation for 5 min, 65 μL of 10% H_3_PO_4_ was added to neutralized the samples. Derivatized samples were transferred to HPLC vials and analyzed immediately.

Separation was achieved using a Luna C18(2) analytical column (100 × 4.6 mm, 5 μm) equipped with a guard column (Phenomenex Ldt., Germany). The mobile phases consisted of 25 mM Na_2_HPO_4_ buffer (pH 7.0) with methanol at a ratio of 90:10 [v/v] (mobile phase A) or 50:50 [v/v] (mobile phase B), respectively. The elution gradient was programmed as follows: 0% B for 1 min, increase to 30% B over 2.5 min, increase to 100% B over 3.5 min, hold 100% B for 3 min, decrease to 0% B over 2 min, and hold at 0% B for 4 min. The flow rate was set to 1.0 mL/min and column temperature was maintained at 28 °C. Thiochromes were detected via fluorescence (Ex 375 nm/ Em 435 nm).

**Figure 2:**
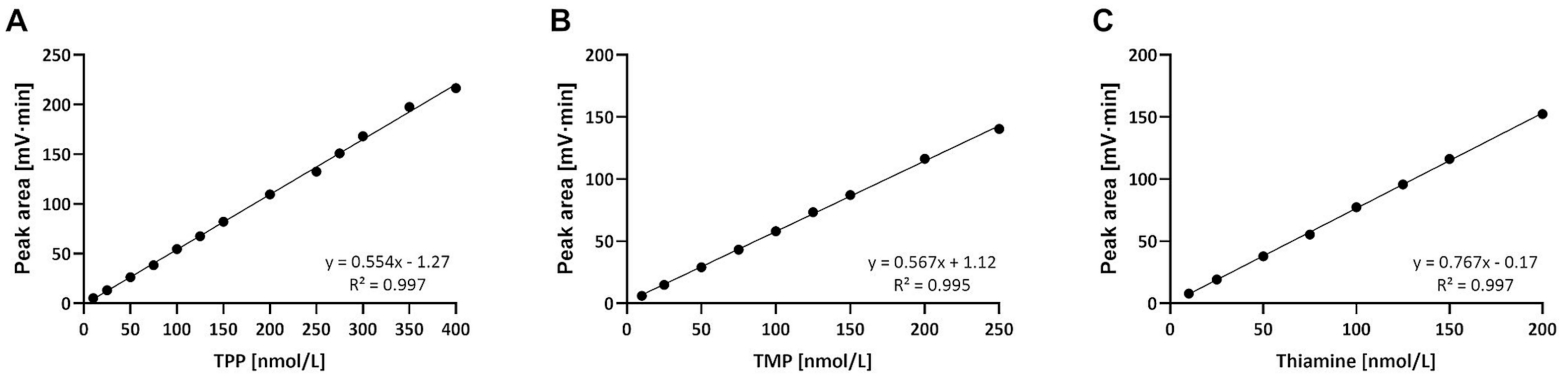
Calibration curves for the quantification of thiamine metabolites by HPLC. Calibration curves of thiamine pyrophosphate [TPP; **(A)**], thiamine monophosphate [TMP; **(B)**], and thiamine **(C)** in saline solution.

Quantification of thiamine and its phosphate esters was achieved with external calibration curves. Calibrator solutions were prepared in saline across concentration ranges of 10–200 nmol/L thiamine hydrochloride (≥99%; Sigma-Aldrich, Germany), 10–250 nmol/L TMP (≥99%; Sigma-Aldrich, Germany) and 10–400 nmol/L TPP (≥95%; Sigma-Aldrich, Germany). Calibration curves for each analyte are presented in Figure 2.

### 2.4 Statistical analysis

All statistical analyses were performed using GraphPad Prism (version 10.3.1).

For comparisons between two groups, data were assessed for normality using the Shapiro–Wilk test and for homogeneity of variances using the F test. When data were normally distributed and variances were homogeneous, an unpaired t-test was applied. For normally distributed data with unequal variances, Welch’s t-test was used. Data that were not normally distributed were analyzed using the Mann–Whitney test.

Comparisons involving more than two groups were performed using one-way analysis of variance (ANOVA) when residuals were normally distributed (Shapiro–Wilk test) and homoscedasticity was confirmed (Brown–Forsythe test), followed by Tukey’s or Dunnett’s multiple comparison test, as appropriate. For data with normally distributed residuals and heterogeneous variances, the Welch’s ANOVA was applied. Non-normally distributed data were analyzed using the Kruskal–Wallis test followed by Dunn’s multiple comparisons test.

Results are presented as mean ± standard deviation (SD). Statistical significance was defined at *p* < 0.05.

## 3 Results

### 3.1 *Drosophila melanogaster* experiments

#### 3.1.1 *Drosophila melanogaster* requires dietary thiamine for larval development

**Figure 3:**
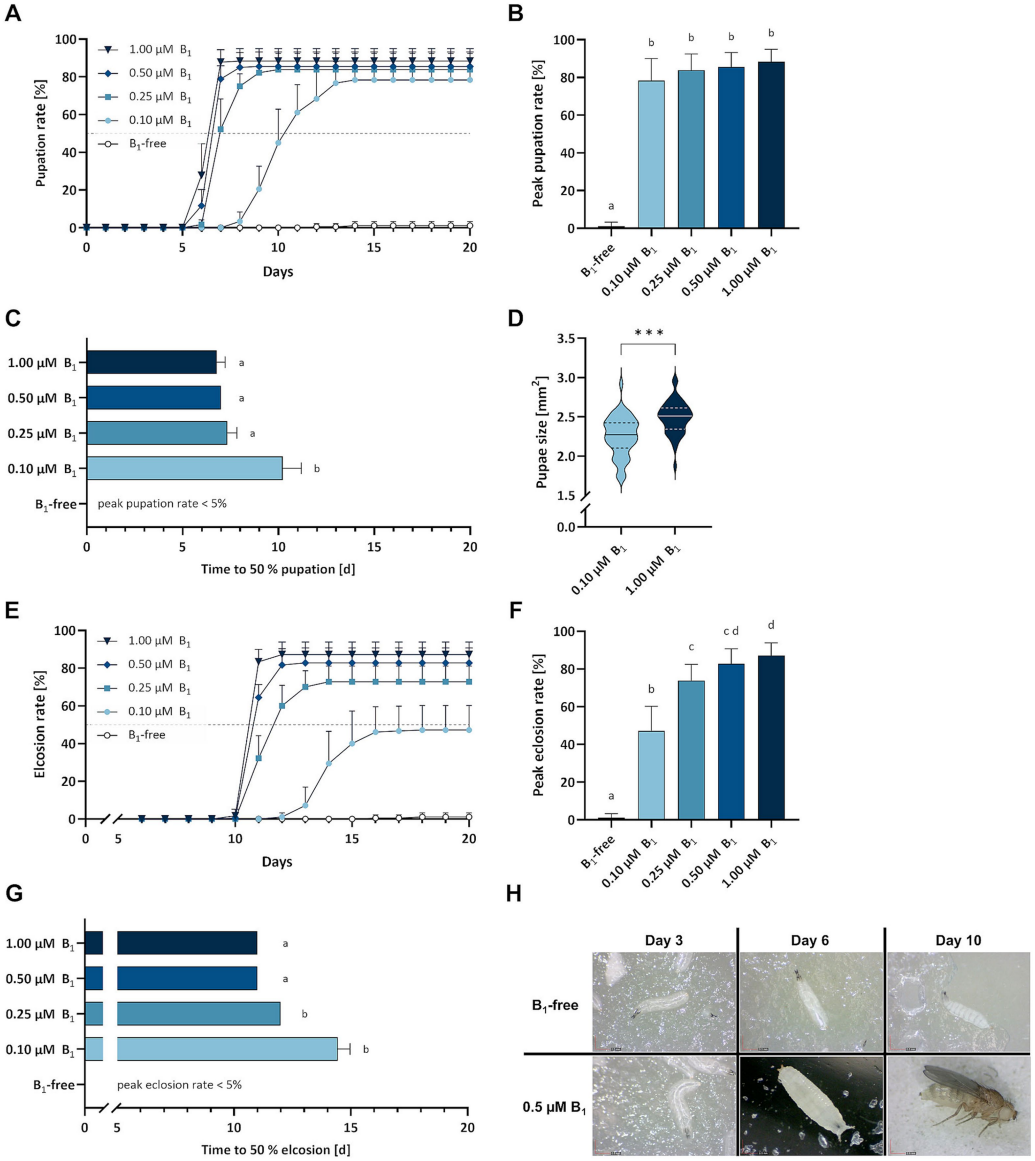
Impact of dietary thiamine on the development of *Drosophila melanogaster*. Twenty synchronized eggs were transferred onto holidic diets containing increasing thiamine (B_1_) concentrations (0–1.0 μM). The development was monitored daily until adulthood. Developmental dynamics until pupation stage **(A)**; corresponding peak pupation rates **(B)**; time to 50% pupation **(C)**; pupae size of larvae raised on diets supplemented with 0.1 μM or 1.0 μM thiamine **(D)**; developmental dynamics until eclosion **(E)**; corresponding peak eclosion rates **(F)**; time to 50% eclosion **(G)**; representative images of the highest developmental stages observed on day 3, 6, and 10 of flies reared on a thiamine-free or 0.5 μM thiamine-supplemented medium (scale bar: 0.5 mm) **(H)**. Data are presented as mean ± standard deviation (SD) from *n* = 3 independent experiments. Each experiment consisted of three replicate vials per condition, each containing 20 eggs, resulting in *N* = 180 eggs per condition. Statistical analyses were conducted using one-way ANOVA (B, F: *p* < 0.001) or Kruskal–Wallis test (C, G: *p* < 0.001), followed by Tukey’s or Dunn’s multiple comparison test, respectively. Groups with development rates <5% were excluded from the timing analyses **(C,G)**. Different small letters indicate significant differences between groups based on post-hoc tests (*p* < 0.05). The Violin plot **(D)** represents the data distribution, median (solid line) and quartiles (dashed lines) from *n* = 3 independent experiments. Each experiment consisted of three replicate vials per condition, with five pupae randomly selected per vial for measurement, resulting in *N* = 45 pupae per condition. Here, statistical analysis was carried out by unpaired t-test (***p < 0.001).

To evaluate the suitability of *D. melanogaster* as a model organism for assessing thiamine (B_1_) bioactivity, we monitored the development from egg to adult across a range of dietary thiamine concentrations (0–1.0 μM) in a holidic thiamine-free medium. Developmental progression was recorded daily, and pupal size was quantified for the lowest and highest supplemented conditions (0.1 μM and 1.0 μM B_1_; Figures 3A–H).

Complete thiamine deprivation caused severe developmental impairment (Figure 3A), reducing the mean peak pupation rate by approximately 99% compared with all tested thiamine-supplemented conditions (Figure 3B). Larvae reared on thiamine-free medium predominantly arrested at the first instar stage and failed to progress further (representative images shown in Figure 3H). Among the thiamine-supplemented groups, mean peak pupation rates did not differ significantly (Figure 3B). However, the time to 50% pupation (TTP_50_), defined as the time required for 50% of surviving larvae to reach the pupal stage, was significantly prolonged in larvae reared on 0.1 μM thiamine (TTP_50_: 10.2 ± 1.0 days), compared with larvae reared on higher thiamine concentrations, for which TTP_50_ ranged from 6.8 ± 0.4 days (1.0 μM thiamine) to 7.3 ± 0.5 days (0.25 μM thiamine), corresponding to an approximately 40–50% prolongation (Figure 3C). This delay was accompanied by reduced pupal size. Pupae from the 0.1 μM thiamine condition were approximately 10% smaller than those from the 1.0 μM condition (Figure 3D).

Although peak pupation rates were comparable among the thiamine-supplemented groups, eclosion rate (Figure 3E) and corresponding peak eclosion rate (Figure 3F) were markedly reduced in the 0.1 μM group. In this group, only 47.2 ± 13.0% of larvae reached the imago stage, compared with 82.8 ± 7.9% and 87.2 ± 6.7% in the 0.5 μM and 1.0 μM thiamine groups, respectively. Analogous to TTP_50_, the time to 50% eclosion (TTE_50_), defined as the time required for 50% of surviving larvae to have eclose, was determined. Consistent with the TTP_50_ results, flies reared on 1.0 μM thiamine reached TTE_50_ more rapidly after 11 days compared with those on 0.1 μM thiamine (14.4 ± 0.5 days; Figure 3G). Of note, this delay is due to the aforementioned slower larval development, as it was not further extended beyond the previously observed TTP_50_ difference between these two dietary thiamine concentrations. Together, these findings demonstrate that *Drosophila’s* developmental dynamics are highly sensitive to thiamine levels, supporting its use as a model for evaluating thiamine bioactivity.

#### 3.1.2 Allithiamine exhibits comparable bioactivity to thiamine in the *Drosophila* development model

**Figure 4:**
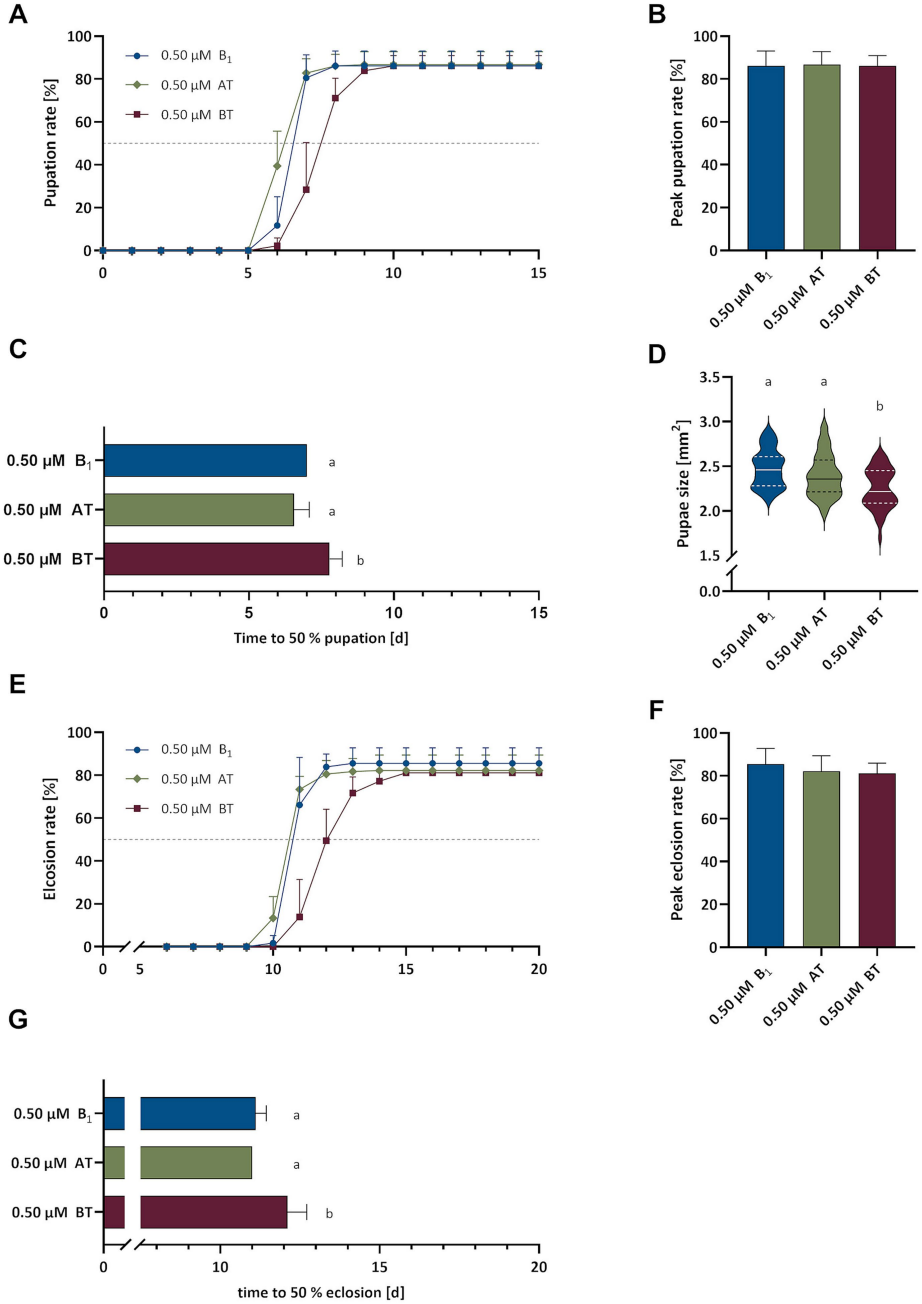
Impact of dietary allithiamine and benfotiamine on the development of *Drosophila melanogaster*. Twenty synchronized eggs were transferred onto holidic diets containing 0.5 μM of either thiamine (B_1_), allithiamine (AT) or benfotiamine (BT). The development was monitored daily until adulthood. Developmental dynamics until pupation stage **(A)**; corresponding peak pupation rates **(B)**; time to 50% pupation **(C)**; pupae size **(D)**; developmental dynamics until eclosion **(E)**; corresponding peak eclosion rates **(F)**; time to 50% eclosion **(G)**. Data are present as mean ± standard deviation (SD) from *n* = 3 independent experiments. Each experiment consisted of three replicate vials per condition, each containing 20 eggs, resulting in *N* = 180 eggs per condition. The Violin plot **(D)** represents the data distribution, median (solid line) and quartiles (dashed lines) from *n* = 3 independent experiments. Each experiment consisted of three replicate vials per condition, with five pupae randomly selected per vial for measurement, resulting in a total of *N* = 45 pupae per condition. Overall group differences were assessed using one-way ANOVA (D: *p* < 0.001; F: *p* = 0.34) or Kruskal–Wallis test (B: *p* = 0.99; C and G: *p* < 0.001), followed by Tukey’s or Dunn’s multiple comparison test, respectively. Different small letters indicate significant differences between groups based on post-hoc tests (*p* < 0.05).

Next, the established *D. melanogaster* development model was employed to evaluate whether allithiamine (AT) exhibits thiamine (B_1_) activity. To this end, developmental progression from egg to adult was determined in flies cultured on a thiamine-free holidic medium supplemented with 0.5 μM of either thiamine, AT or benfotiamine (BT; Figures 4A–G). The synthetic thiamine analogue BT served as a lipophilic reference compound.

AT supplementation did not differ from thiamine supplementation in peak pupation rate, TTP_50_, or pupal size (Figures 4B–D). Likewise, no significant differences were observed between these two groups in peak eclosion rate or TTE_50_ (Figures 4F, G).

When BT was administered, neither peak pupation rate nor peak eclosion rate differed significantly from those observed in the thiamine- or AT-supplemented groups (Figures 4B, F). In contrast, flies reared on BT-supplemented medium reached TPP_50_ approximately 0.7 and 1.2 days later than flies reared on thiamine- and AT-supplemented medium, respectively (Figure 4C). This reflects a developmental prolongation of approximately 10% and 18%, respectively. A similar delay was observed for TTE_50_. Whereas flies reared on thiamine- and AT-supplemented medium reached TTE_50_ after approximately 11 days, BT-supplemented flies reached TTE_50_ approximately 1 day later, representing a prolongation of approximately of 9% (Figure 4G). In addition, pupae in the BT-supplemented group were significantly smaller than pupae from thiamine- and AT-supplemented medium, with an average size of 2.25 ± 0.22 mm^2^ compared to 2.48 ± 0.21 mm^2^ in the thiamine group and 2.40 ± 0.24 mm^2^ in the AT group, corresponding to reductions of approximately 9% and 6%, respectively (Figure 4D).

#### 3.1.3 Allithiamine supplementation leads to higher larval thiamine pyrophosphate levels than thiamine supplementation

**Figure 5:**
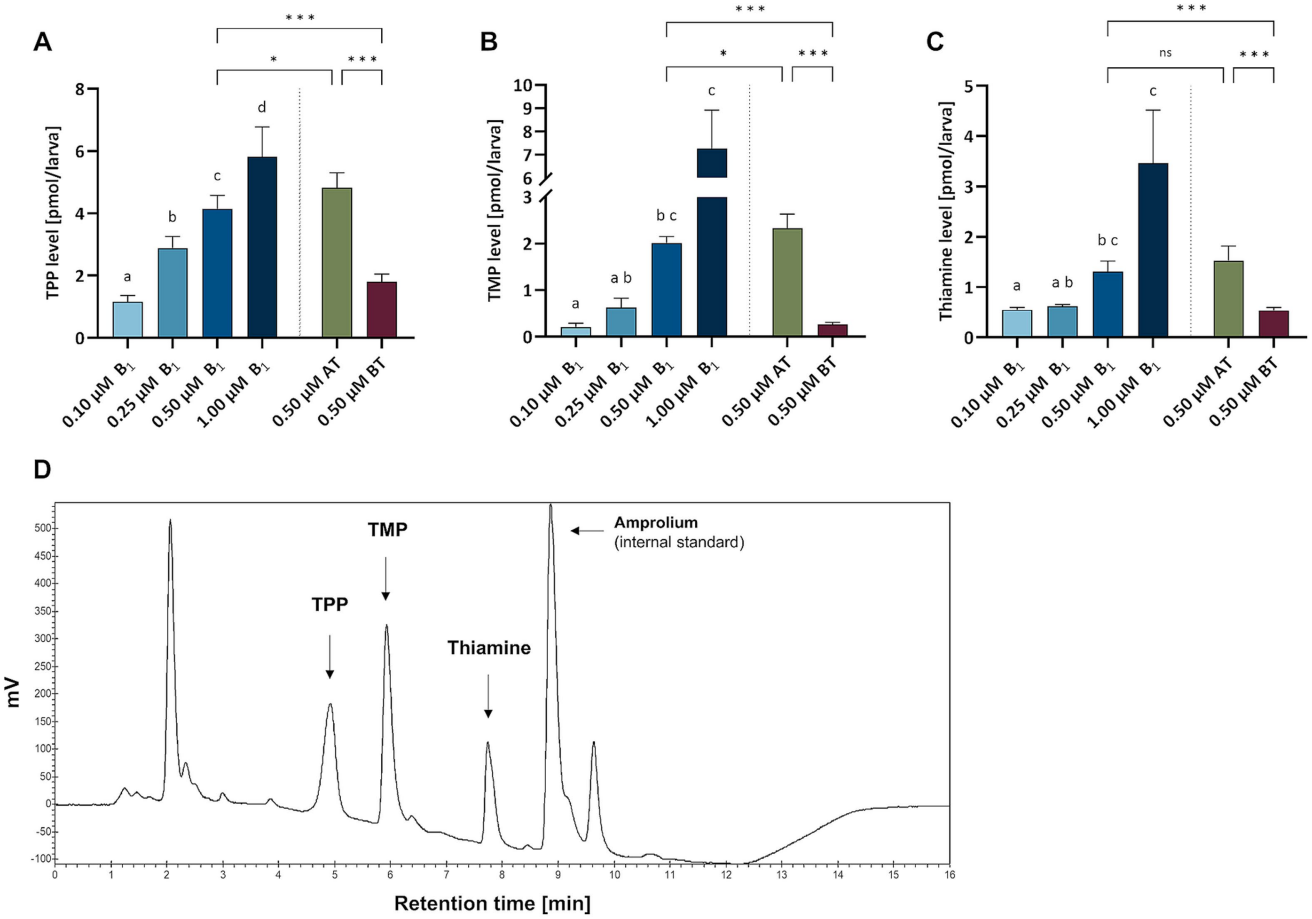
Levels of thiamine pyrophosphate (TPP), thiamine monophosphate (TMP) and free thiamine in wandering third instar *Drosophila melanogaster* larvae. Approximately 50 synchronized eggs were placed on holidic thiamine-free diets supplemented with thiamine (B_1_; 0–1.0 μM) or 0.5 μM allithiamine (AT) or benfotiamine (BT). TPP **(A)**, TMP **(B)**, and free thiamine **(C)** levels were quantified in wandering larvae using pools of five larvae per sample by HPLC analysis. **(D)** Representative chromatogram of thiamine metabolites in larval homogenate recorded at Ex 375 nm/Em 435 nm. Bars represent the mean ± standard deviation (SD) from *n* = 3 independent experiments. Each experiment consisted of two replicate vials per condition, from which five larvae per vial were pooled for analysis, resulting in *N* = 30 larvae per condition. Differences among the thiamine-supplemented groups and among the 0.5 μM-supplemented groups were statistical evaluated by two separate one-way ANOVAs (both *p* < 0.001) with Tukey’s multiple comparisons test. Different small letters indicate significant differences among thiamine-supplemented groups (*p* < 0.05), while asterisks denote significant differences between the 0.5 μM-supplemented groups (ns: not significant; *p < 0.05; ***p < 0.001).

Developmental parameters were complemented by quantifying thiamine (B_1_) metabolites in wandering L3 using HPLC (Figures 5A–D). A representative chromatogram of vitamin B_1_ metabolites in *Drosophila* L3 is shown in Figure 5D.

Levels of the active co-factor thiamine pyrophosphate (TPP) increased in a dose-dependent manner with increasing dietary thiamine supplementation. Larvae reared on the 0.1 μM thiamine diet exhibited the lowest TPP levels (1.16 ± 0.20 pmol/larva), whereas those maintained on the 1.0 μM thiamine diet showed approximately 5-fold higher levels (5.82 ± 0.96 pmol/larva; Figure 5A).

Thiamine monophosphate (TMP) and free thiamine levels showed a similar increase. Larvae reared on 0.1 μM thiamine medium exhibited TMP and free thiamine levels of 0.21 ± 0.08 pmol/larva and 0.55 ± 0.05 pmol/larva, respectively, whereas 1.0 μM thiamine-supplemented larvae reached TMP levels of 7.26 ± 1.65 pmol/larva and free thiamine levels of 3.47 ± 1.05 pmol/larva (Figures 5B, C).

At equivalent supplementation levels (0.5 μM), allithiamine (AT)-treated larvae displayed significantly higher TPP and TMP levels (4.82 ± 0.48 pmol/larva and 2.33 ± 0.30 pmol/larva, respectively) compared to thiamine-treated larvae, which reached TPP and TMP levels of 4.15 ± 0.42 pmol/larva and 2.01 ± 0.14 pmol/larva, respectively (Figures 5A, B). In contrast, no difference was observed in free thiamine levels between those groups with 1.53 ± 0.29 pmol/larva (AT group) vs. 1.31 ± 0.21 pmol/larva (thiamine group; Figure 5C).

In contrast, 0.5 μM BT supplementation resulted in substantially lower TPP, TMP and free thiamine levels (1.80 ± 0.25 pmol/larva, 0.27 ± 0.04 pmol/larva and 0.54 ± 0.05 pmol/larva, respectively) when compared with the corresponding AT- and thiamine-treated larvae (Figures 5A–C).

### 3.2 *Mus musculus* experiments

#### 3.2.1 Thiamine and allithiamine supplementation show comparable body weight gain, food intake and liver indices

Mice supplemented with thiamine (B_1_) or AT showed comparable body weight gain and food intake at both day 1 and day 7 of supplementation, resulting in similar thiamine or AT intake. Furthermore, the hepatosomatic index [calculated by body weight (g)/ liver weight (g)] did not differ significantly between the two treatment groups at either supplementation periods (Table 4).

**Table 4:** Impact of thiamine deprivation, followed by supplementation with either thiamine (B_1_) or allithiamine (AT) for 1 or 7 days, on body weight gain, food intake and liver weight in mice.

Parameters	Thiamine-free (B_1_-free) group
Total body weight gain (g)	4.65 ± 1.58
Total food intake (g)	24.72 ± 2.25
Total B_1_/AT intake (nmol)	—
Hepatosomatic index	6.34 ± 0.63

Parameters	Thiamine (B_1_) groups	Allithiamine (AT) groups	*p*-value
After 1 day supplementation	B_1_-d1	AT-d1	B_1_-d1 vs AT-d1
Total body weight gain (g)	5.72 ± 2.11	5.22 ± 1.59	0.60
Total food intake (g)	30.02 ± 1.08	29.27 ± 1.07	0.19
Total B_1_/AT intake (nmol)	32.96 ± 4.58	34.34 ± 2.59	0.47
Hepatosomatic index	6.31 ± 0.44	6.40 ± 0.50	0.72
After 7 days supplementation	B_1_-d7	AT-d7	B_1_-d7 vs AT-d7
Total body weight gain (g)	6.19 ± 2.41	6.51 ± 1.32	0.74
Total food intake (g)	47.51 ± 1.79	46.47 ± 2.67	0.37
Total B_1_/AT intake (nmol)	212.5 ± 12.84	209.99 ± 17.89	0.72
Hepatosomatic index	5.74 ± 0.41	5.33 ± 0.67	0.16

Body weight gain was measured on weekdays, and food intake was recorded daily. The total thiamine and AT intake were calculated from food consumption. The hepatosomatic index (liver weight/ body weight) was determined at the end of the experiment. Data are given as mean ± standard deviation (SD) for *n* = 8 mice per group. Differences of pairwise comparisons at day 1 and day 7 of supplementation (B_1_-d1 vs. AT-d1; B_1_-d1 vs. AT-d7) were performed using unpaired *t*-test, Welch’s test, or Mann–Whitney test as appropriate.

#### 3.2.2 Thiamine pyrophosphate level increase with thiamine and allithiamine supplementation

**Figure 6:**
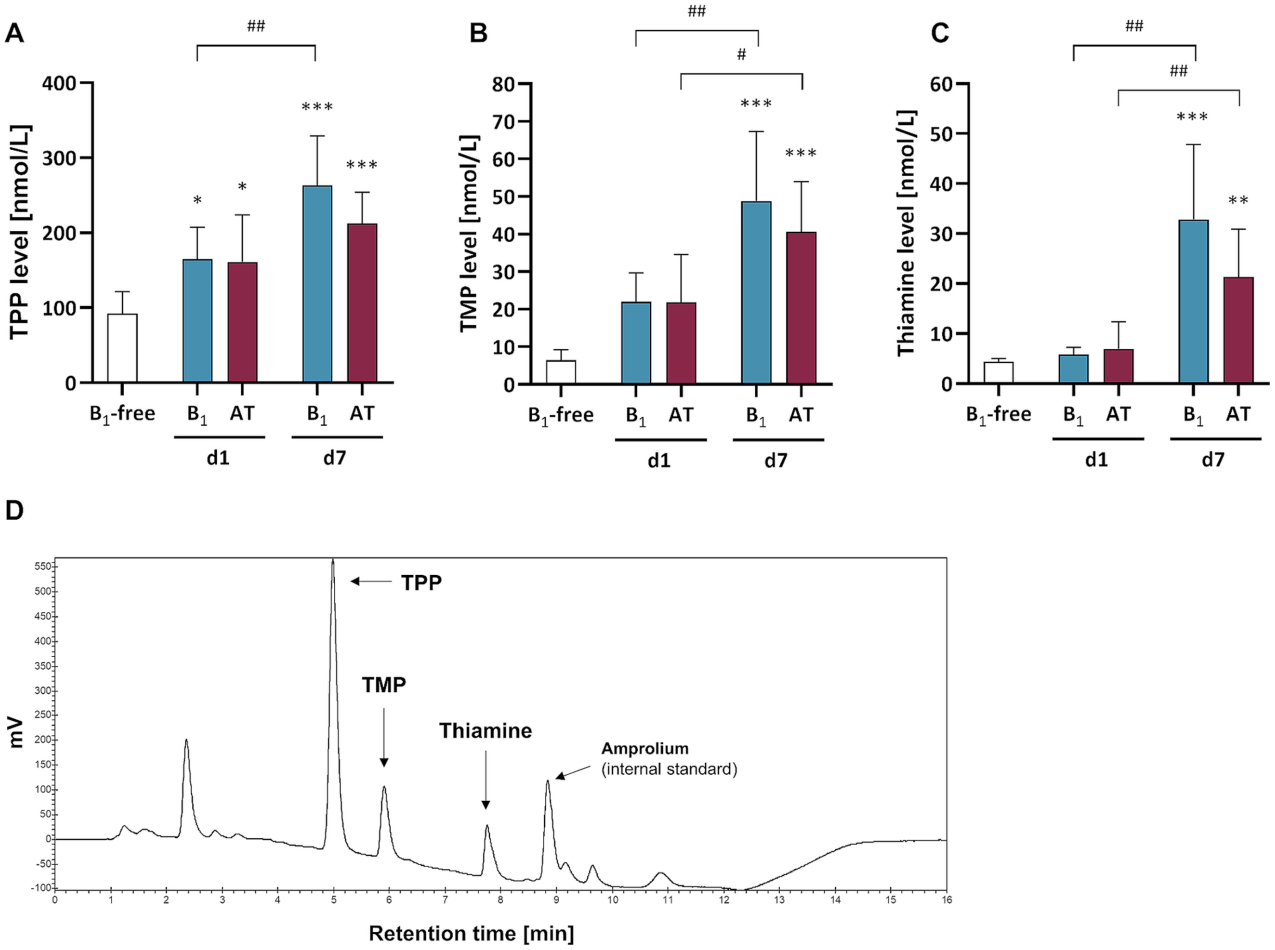
Thiamine pyrophosphate (TPP), thiamine monophosphate (TMP), and free thiamine level after 1 week of thiamine deprivation (B_1_-free), followed by supplementation with either thiamine (B_1_) or allithiamine (AT) for 1 or 7 days in male mice. TPP **(A)**, TMP **(B)**, and free thiamine **(C)** levels were quantified in whole blood by HPLC analysis. Representative chromatogram of thiamine metabolites in murine whole blood recorded at Ex 375 nm/Em 435 nm **(D)**. Data are presented as mean ± standard deviation (SD) for *n* = 8 mice per group. Differences of the supplemented groups compared to the thiamine-free group were analyzed by either one-way ANOVA (A: *p* < 0.001) followed by Dunnett’s multiple comparisons test or Kruskal–Wallis test (B, C: *p* < 0.001) followed by Dunn’s multiple comparisons test. Significant differences based on post-hoc tests are indicated by asterisks (*p < 0.05; **p < 0.01; ***p < 0.001). Additional pairwise comparisons (B_1_-d1 vs. AT-d1; B_1_-d7 vs. AT-d7; B_1_-d1 vs. B_1_-d7; AT-d1 vs. AT-d7) were performed using unpaired *t*-test, Welch’s *t*-test or Mann–Whitney test as appropriate. Hash symbols denote significant differences as indicated (^#^*p* < 0.05; ^##^*p* < 0.01).

The thiamine (B_1_) status of mice was assessed by HPLC analysis of whole blood after 1 week of thiamine deprivation with or without a subsequent supplementation with either thiamine or AT for either 1 or 7 days (Figures 6A–D). A representative chromatogram of thiamine metabolites in murine whole blood is shown in Figure 6D.

At the end the thiamine-free period, mice exhibited a mean TPP level of 92.3 ± 29.2 nmol/L. After 1 day of supplementation, TPP levels increased significantly to 164.9 ± 42.7 nmol/L in the thiamine group and to 161.2 ± 62.5 nmol/L in the AT group. Supplementation for 7 days resulted in a further increase, with TPP concentrations of 263.3 ± 65.8 nmol/L in thiamine-treated mice and 212.4 ± 41.9 nmol/L in AT-treated mice (Figure 6A).

TMP and free thiamine levels of thiamine-depleted mice were 6.44 ± 2.78 nmol/L and 4.38 ± 0.66 nmol/L, respectively (Figures 6B, C). A slightly but non-significant increase in both metabolites was observed after 1 day supplementation with either thiamine or AT compared with the thiamine-free group. After 7 days of supplementation, TMP and free thiamine levels increased significantly to 48.8 ± 18.5 nmol/L and 32.8 ± 15.0 nmol/L, respectively in thiamine-treated mice and to 40.6 ± 13.4 nmol/L and 21.3 ± 9.5 nmol/L, respectively in AT-treated mice compared with thiamine-depleted animals (Figures 6B, C).

#### 3.2.3 Seven-day thiamine supplementation increases intestinal Slc19a2, but not Slc19a3 mRNA levels

**Figure 7:**
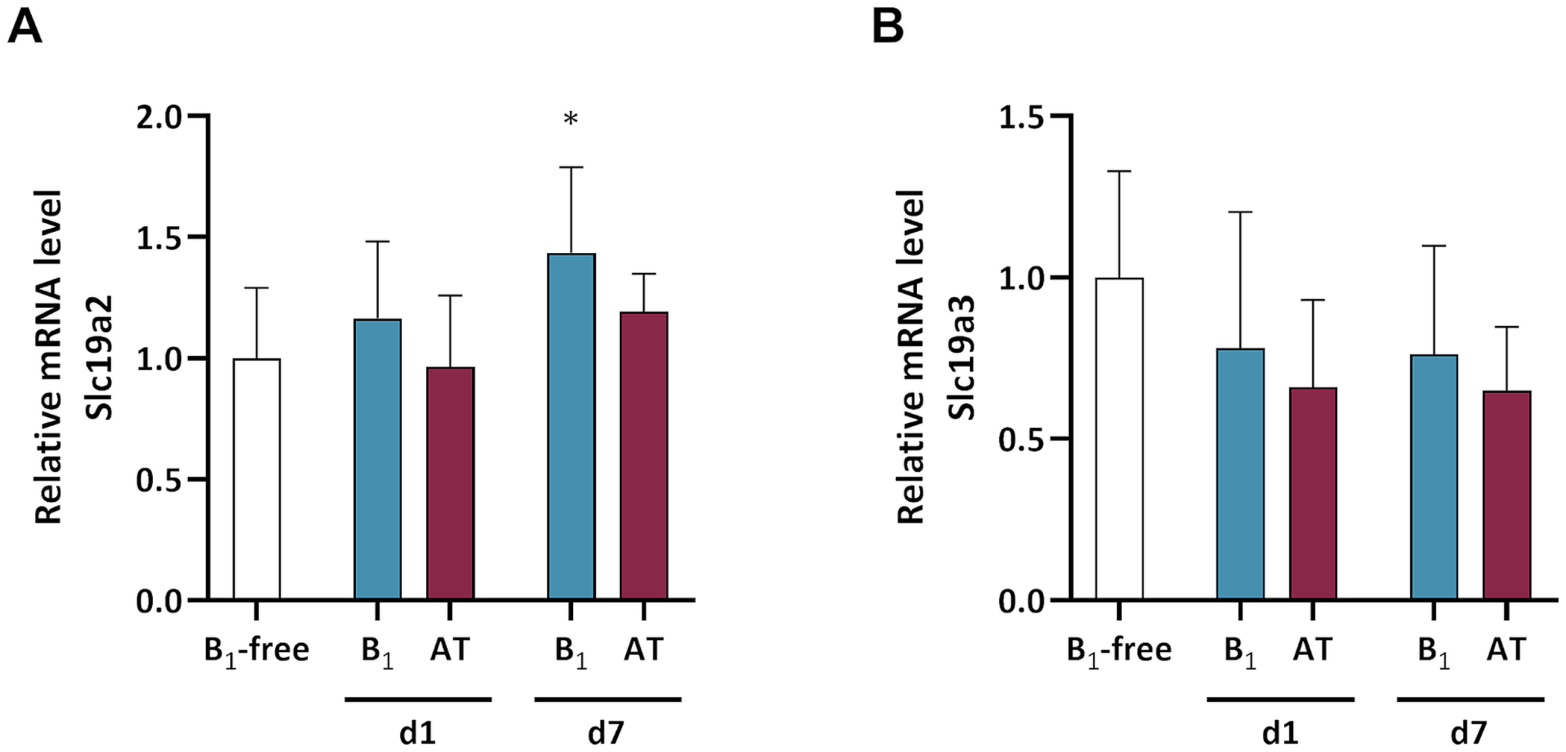
Impact of 1 week of thiamine deprivation (B_1_-free), followed by supplementation with either thiamine (B_1_) or allithiamine (AT) for 1 or 7 days, on mRNA levels of thiamine transporter genes in the intestinal mucosa of mice. Transcript levels of Slc19a2 **(A)** and Slc19a3 **(B)** were analyzed using quantitative reverse transcription real-time polymerase chain reaction (qRT-PCR). Data were normalized to 18sRNA and *β*-actin and related to the thiamine-free group. Data are given as the mean ± standard deviation (SD) for *n* = 7–8 mice per group. Differences of the supplemented groups compared to the thiamine-free group were analyzed by one-way ANOVA (Slc19a2: *p* = 0.03; Slc19a3: *p* = 0.23) followed by Dunnett’s multiple comparisons test. Significant differences based on post-hoc tests are indicated by asterisks (*p < 0.05). Additional pairwise comparisons (B_1_-d1 vs. AT-d1; B_1_-d7 vs. AT-d7; B_1_-d1 vs. B_1_-d7; AT-d1 vs. AT-d7) were performed using unpaired *t*-test.

The mRNA levels of genes encoding key proteins involved in intestinal thiamine transport (Slc19a2 and Slc19a3) were assessed in murine intestinal mucosa by qRT-PCR (Figures 7A,B). Relative Slc19a2 mRNA levels were significantly increased by 1.34-fold in mice supplemented with thiamine for 7 days compared with thiamine-depleted mice, but did not differ significantly from mice receiving AT for 7 days. No significant changes in Slc19a2 mRNA levels were observed in the other supplemented groups (Figure 7A).

In contrast, Slc19a3 mRNA levels were not significantly altered throughout the experimental period. However, mean transcript levels were numerically lower in supplemented mice compared with thiamine-depleted mice (Figure 7B).

#### 3.2.4 Thiamine and allithiamine comparably increase PDHA1 phosphorylation after 7 days of supplementation

**Figure 8:**
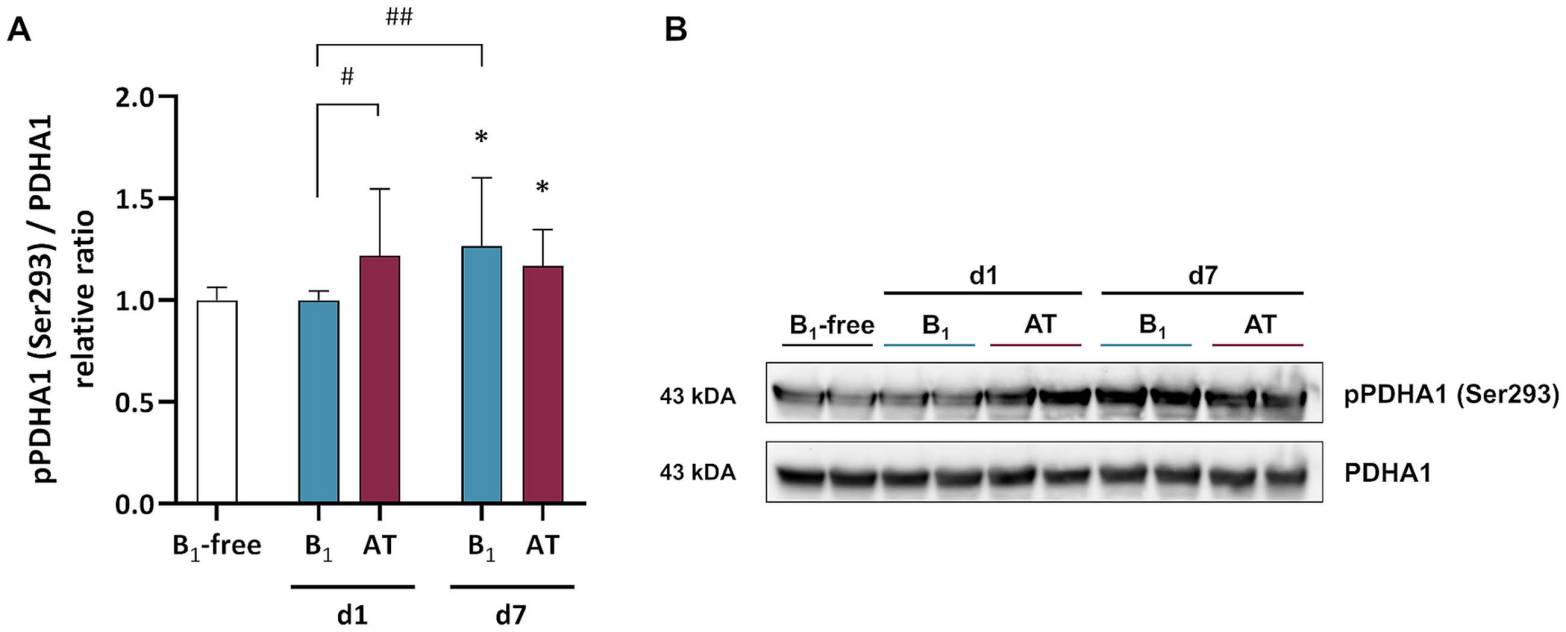
Impact of 1 week of thiamine deprivation (B_1_-free), followed by supplementation with either thiamine (B_1_) or allithiamine (AT) for 1 or 7 days, on hepatic protein expression levels of phosphorylated pyruvate dehydrogenase E1 subunit alpha (pPDHA1) and total PDHA1 in mice. Protein expression levels were analyzed by Western blot and the ratio of pPDHA1 and total PDHA1 was calculated for each sample. Relative ratio of pPDHA1 to total PDHA1 **(A)** and a representative blot **(B)** are shown. Protein expression was normalized to total protein load per lane and a pooled reference sample. Data are presented as mean ± standard deviation (SD) for *n* = 8 mice per group. Differences between supplemented groups and the thiamine-free group were analyzed using the Kruskal–Wallis test (*p* < 0.001) followed by Dunn’s multiple comparisons test. Significant differences based on post-hoc tests are indicated by asterisks (*p < 0.05). Additional pairwise comparisons (B_1_-d1 vs. AT-d1; B_1_-d7 vs. AT-d7; B_1_-d1 vs. B_1_-d7; AT-d1 vs. AT-d7) were performed using Mann–Whitney test. Hash symbols denote significant differences as indicated (^#^*p* < 0.05; ^##^*p* < 0.01).

Given the increasing TPP levels over the supplementation period, the phosphorylation status of the thiamine-dependent pyruvate dehydrogenase E1 subunit alpha (PDHA1) was investigated by measuring the protein expression of phosphorylated PDHA1 (Ser293) and total PDHA1 by western blotting (Figures 8A,B). PDHA1 is a subunit of the E1 component of the pyruvate dehydrogenase complex (PDC) and catalyzes the oxidative decarboxylation of pyruvate, the first step in the overall conversion of pyruvate to acetyl-CoA by the PDC (26).

The pPDHA1/PDHA1 ratio was significantly increased after 7 days of supplementation, by 1.27-fold in the thiamine (B_1_) group and 1.17-fold in the AT group, compared with the thiamine-free group, indicating enhanced PDHA1 phosphorylation. Although there was no significant difference of thiamine- and AT-treated mice after 1 day of supplementation compared with the thiamine-free mice, AT-treated mice showed a 1.22-fold higher pPDHA1/PDHA1 ratio in contrast to thiamine-treated mice (Figure 8).

#### 3.2.5 Thiamine and allithiamine supplementation selectively increase hepatic ATP5A protein levels

**Figure 9:**
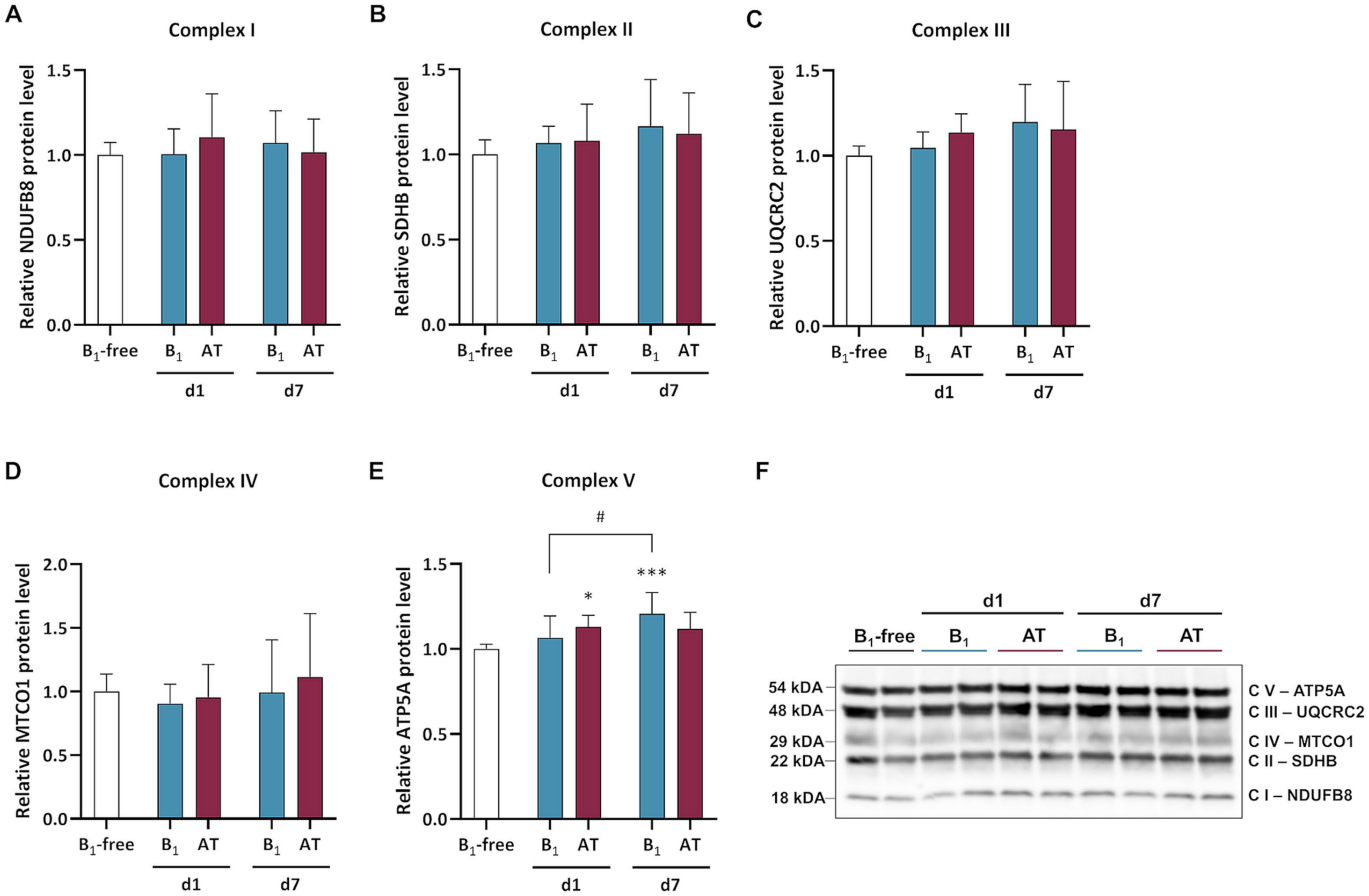
Impact of 1 week of thiamine deprivation (B_1_-free), followed by supplementation with either thiamine (B_1_) or allithiamine (AT) for 1 or 7 days, on hepatic protein expression levels of representative subunits of mitochondrial oxidative phosphorylation (OXPHOS) complexes I–V in mice. Protein expression levels were analyzed by Western blot. Relative protein levels of NADH dehydrogenase [ubiquinone] 1 beta subcomplex subunit 8 (NDUFB8; complex I) **(A)**, succinate dehydrogenase subunit B (SDHB; complex II) **(B)**, ubiquinol-cytochrome c reductase complex core protein 2 (UQCRC2; complex III) **(C)**, cytochrome c oxidase subunit 1 (MTCO1; complex IV) **(D)**, ATP synthase F1 subunit alpha (ATP5A; complex V) **(E)** and a representative blot **(F)** are shown. Protein expression was normalized to total protein load per lane and to a pooled reference sample. Data are presented as mean ± standard deviation (SD) for *n* = 8 mice per group. Differences between supplemented groups and the thiamine-free group were analyzed by one-way ANOVA (ATP5A: *p* = 0.002) followed by Dunnett’s multiple comparisons test, Kruskal–Wallis test (SDHB: *p* = 0.52; UQCRC2: *p* = 0.12; MTCO1: *p* = 0.83) or Welch’s ANOVA (NDUFB8: *p* = 0.76). Significant differences based on post-hoc tests are indicated by asterisks (*p < 0.05; ***p < 0.001). Additional pairwise comparisons (B_1_-d1 vs. AT-d1; B_1_-d7 vs. AT-d7; B_1_-d1 vs. B_1_-d7; AT-d1 vs. AT-d7) were performed using t-test or Mann–Whitney test as appropriate. Hash symbols denote significant differences as indicated (^#^*p* < 0.05).

As thiamine is required for several enzymes involved in cellular energy metabolism, mitochondrial oxidative phosphorylation (OXPHOS) complex protein levels were assessed by Western blot analysis using representative subunits of OXPHOS complexes I–V (Figures 9A–F). No significant differences were observed for the subunits of complexes I–IV throughout the experimental period (Figures 9A–D). In contrast, relative protein levels of ATP synthase F1 subunit alpha (ATP5A), a subunit of complex V, were selectively increased following supplementation (Figure 9E). After 1 day of supplementation, AT-treated mice exhibited a significant 1.13-fold increase in ATP5A protein levels compared with thiamine-free mice, whereas no significant alteration was observed in thiamine-supplemented mice at this time point. After 7 days of supplementation, ATP5A levels in AT-treated mice showed a non-significant increase compared with thiamine-free mice (1.12-fold; *p* = 0.06), while thiamine-supplemented mice exhibited a significant 1.21-fold increase (Figure 9E).

#### 3.2.6 Hepatic mRNA levels of thiamine-related and metabolic genes did not differ significantly from thiamine-free mice during the experimental period

**Figure 10:**
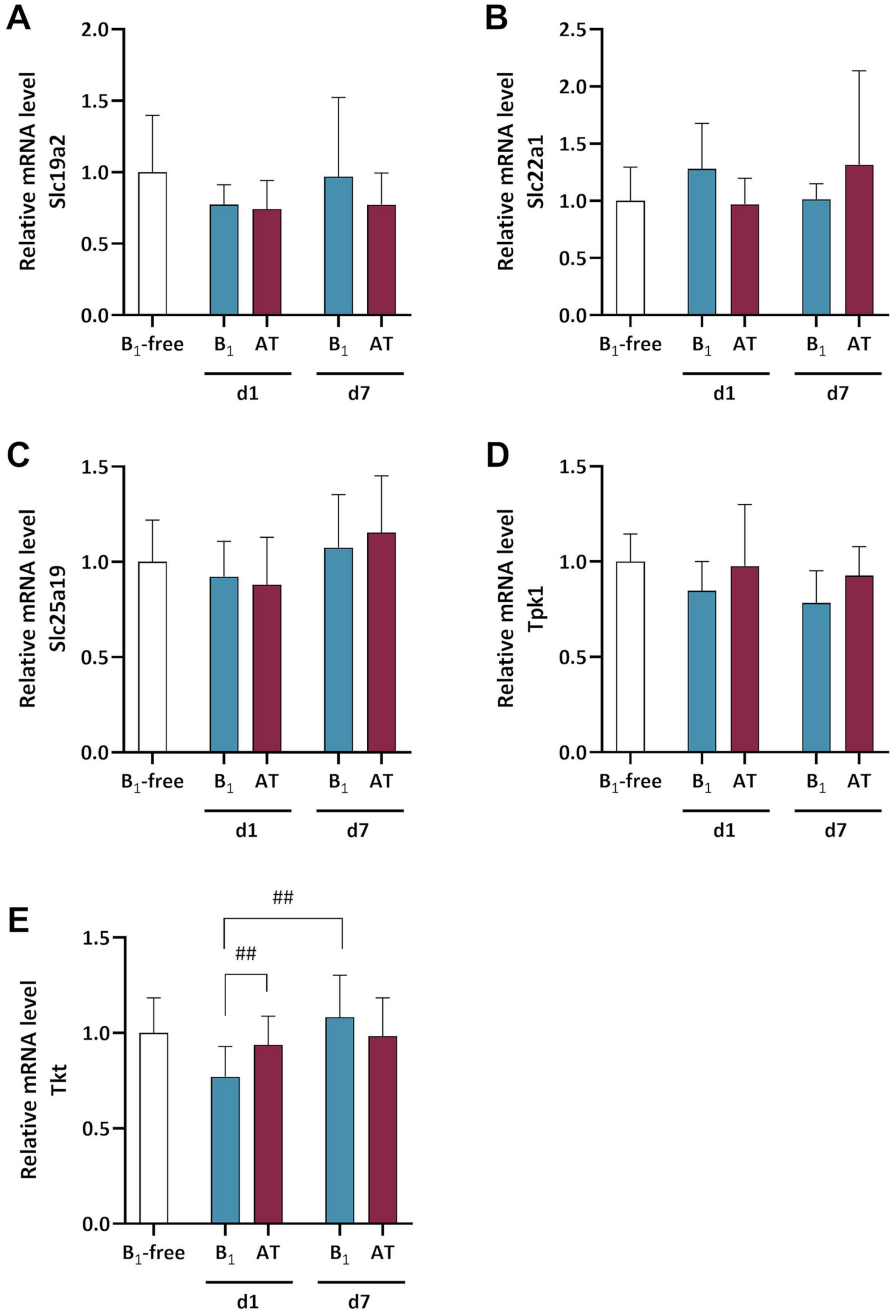
Impact of 1 week of thiamine deprivation (B_1_-free), followed by supplementation with either thiamine (B_1_) or allithiamine (AT) for 1 or 7 days, on hepatic mRNA levels of thiamine related genes in mice. Transcript levels were analyzed using quantitative reverse transcription real-time polymerase chain reaction (qRT-PCR). Relative mRNA expression of Slc19a2 **(A)**, Slc22a1 **(B)**, Slc25a19 **(C)**, Tpk1 **(D)** and Tkt **(E)** are shown. The mRNA levels were normalized to 18sRNA and β-actin and related to the thiamine-free group. Data are given as the mean ± standard deviation (SD) for n = 8 mice per group. Differences of the supplemented groups compared to the thiamine-free group were analyzed by one-way ANOVA (Slc25a19: *p* = 0.20; Tpk1: *p* = 0.19; Tkt: *p* = 0.03) followed by Dunnett’s multiple comparisons test or by Kruskal-Wallis test (Slc19a2: *p* = 0.59, Slc22a1: *p* = 0.32). Additional pairwise comparisons (B_1_-d1 vs. AT-d1; B_1_-d7 vs. AT-d7; B_1_-d1 vs. B_1_-d7; AT-d1 vs. AT-d7) were performed using unpaired *t*-test, Welch’s *t*-test or Mann–Whitney test as appropriate. Hash symbols denote significant differences as indicated (^##^*p* < 0.01).

Relative hepatic mRNA levels of genes encoding proteins involved in thiamine transport (Slc19a2, Slc22a1 and Slc25a19) and its conversion to its active cofactor TPP (thiamine pyrophosphokinase 1, Tpk1) did not differ significantly between supplemented groups and thiamine-free mice (Figures 10A–D). Hepatic Slc19a3 mRNA levels were additionally assessed. However, transcript levels remained below the detection limit, presumably due to the very low intrinsic expression of Slc19a3 in the liver, as previously reported (27, 28).

In addition, hepatic mRNA levels of the thiamine-dependent enzyme transketolase (Tkt) were analyzed. After 1 day of supplementation, AT-treated mice exhibited significantly higher Tkt mRNA levels than thiamine-treated mice, corresponding to an approximately 1.2-fold increase (Figure 10E). This difference was no longer observed after 7 days, as Tkt mRNA levels in thiamine-treated mice increased significantly over time, by approximately 1.4-fold. However, none of the supplemented groups differed significantly from thiamine-free mice at the respective time points (Figure 10E).

## 4 Discussion

In the present study, we established a complementary *in vivo* framework combining *Drosophila melanogaster* and laboratory mice to comparatively assess the bioactivity of thiamine and its lipophilic derivative allithiamine. Our data demonstrate that thiamine availability is a crucial factor in *Drosophila* larval development, as allowing us to develop a sensitive model for determining thiamine bioactivity at whole-organism level. We thus proved that allithiamine is functionally comparable to thiamine in terms of both development and metabolism. Although one might assume that allithiamine could have an advantage over thiamine regarding bioavailability due to its different chemical properties, our data suggest that this superiority does not manifest under physiological conditions when both compounds are administered in equimolar doses.

We demonstrate that in *D. melanogaster*, an adequate dietary thiamine intake is essential for successful development beyond the first-instar larval stage. This also implies, that maternal supply of thiamine, which most likely enables embryonic development, is limited. The essential role of thiamine in *Drosophila* development has been reported previously (17). Consistent with our findings, Sannino et al. (17) showed that axenic larvae reared on a thiamine-free medium failed to survive and died prior to pupation, whereas dietary thiamine supplementation restored normal development. Moreover, they reported that approximately 20% of non-axenic flies reached adulthood despite the absence of dietary thiamine and attributed their findings to a specific thiamine provisioning gut microbiota member. In contrast, survival of non-axenic flies in our study more closely resembled the axenic phenotype described by Sannino et al. (17), suggesting that thiamine-producing microbial taxa may be less abundant in our laboratory stock. Such inter-laboratory variation in *Drosophila* microbiome composition has been reported previously (29) and underscores the importance to account for microbiota when using *Drosophila* as a developmental bioassay for thiamine bioactivity.

By directly quantifying changes in thiamine status in responds to varying dietary thiamine doses and linking these biochemical readouts to developmental and phenotypic outcomes, our results highlight the utility of *D. melanogaster* as a whole-organism model for assessing thiamine bioactivity. This integrative approach provides a distinct advantage over traditional microbiological assays, which rely on indirect, growth-related turbidity measurements and may respond nonspecifically to thiamine precursors, degradation products, or fragments (30). In contrast, the *Drosophila* model enables simultaneous evaluation of bioavailability, metabolic conversion to active cofactors, and developmental and phenotypic effects, providing a more comprehensive and biologically relevant assessment of thiamine activity *in vivo*.

Using our established development model, we demonstrate, to the best of our knowledge for the first time, that dietary allithiamine fully restores developmental impairments caused by thiamine deficiency to the same extent as an equimolar dose of thiamine. This indicates that allithiamine provides sufficient thiamine bioactivity to sustain normal development at the whole-organism level. Extending these findings, our murine repletion model revealed overall comparable metabolic responses to equimolar supplementation with allithiamine or thiamine under physiologically relevant conditions. Previous investigations directly comparing allithiamine and thiamine bioactivity are limited and largely date back several decades (31, 32), whereas more recent studies have primarily focused on the therapeutic potential of allithiamine rather than its nutritional equivalence (33, 34). Despite their relatively simple experimental design, often relying on small group sizes and gross phenotypic endpoints such as weight gain and survival, these early studies reported outcomes broadly consistent with our findings. Lilly et al. (31) observed similar growth recovery in thiamine-deficient rats following supplementation with either thiamine or allithiamine, suggesting dietary functional interchangeability. Likewise, de Renzo et al. (32) reported comparable survival and growth outcomes in thiamine-depleted rats following thiamine or allithiamine supplementation, alongside a modest increase in hepatic total thiamine content in allithiamine-supplemented animals. This is in good accordance with our observations of slightly higher tissue thiamine levels in *Drosophila* larvae reared on allithiamine supplemented medium. In contrast, murine whole blood thiamine levels were comparable between thiamine- and allithiamine-supplemented mice, suggesting allithiamine may preferentially promotes thiamine tissue distribution rather than increasing circulating thiamine levels.

By combining developmental and phenotypic readouts in *Drosophila* with targeted biochemical and metabolic assessments in mice, our work provides a systematic and cross-species *in vivo* framework for evaluating thiamine vitamer bioactivity. This dual-model strategy enhances biological robustness, mitigates model-specific bias, and increases translational relevance by demonstrating conserved responses across phylogenetically distinct systems. Collectively, our findings position allithiamine as a biologically active vitamer *in vivo* and provide a robust experimental framework for future studies addressing vitamer-specific kinetics, regulation, and tissue distribution.

Administration of benfotiamine to *Drosophila* resulted in restored peak pupation and eclosion rates to levels comparable to those observed with thiamine and allithiamine supplementation. However, benfotiamine-treated larvae displayed delayed developmental timing, reduced pupal size and markedly lower tissue thiamine level, suggesting slower or less effective bioactivation of benfotiamine. This contrasts with findings from mammalian studies, in which benfotiamine supplementation increases blood and tissue thiamine level (35–37), indicating potential species-specific differences in metabolic processing. Unlike allithiamine, benfotiamine requires intestinal dephosphorylation by alkaline phosphatases prior to entering the circulation as S-benzoylthiamine, followed by esterase-mediated hydrolysis or glutathione-dependent cleavage release to free thiamine (35, 38). Although *Drosophila* possesses the enzymatic machinery required for these reactions (39–41), our data suggest that one or more conversion steps may be rate-limiting in the fly, resulting in reduced functional thiamine availability. While elucidating the metabolism of benfotiamine in *Drosophila* was not a primary objective, this observation represents a relevant secondary finding that warrants further investigation.

In thiamine-deficient mice, blood thiamine levels increased following supplementation with either thiamine and allithiamine. Despite this systemic increase, intestinal thiamine transporter gene expression exhibited only limited responsiveness, characterized by a modest increase in Slc19a2 mRNA levels after 7 days of thiamine supplementation, whereas Slc19a3 transcript levels remained unchanged. These findings suggest that short-term thiamine depletion followed by repletion for up to 1 week exerts only minor effects on intestinal thiamine transporter expression at transcriptional level under the conditions tested. This contrasts with earlier findings reporting unchanged Slc19a2 mRNA levels, but markedly increased Slc19a3 mRNA levels in mice subjected to prolonged thiamine deprivation compared with control mice receiving 6 mg thiamine/kg diet (42). The discrepancy may therefore reflect differences in the duration and severity of thiamine depletion, the timing of analysis, or compensatory adaptation during prolonged deficiency.

In thiamine-deficient mice, we further observed an increased pPDHA1/PDHA1 ratio following supplementation with thiamine or allithiamine, suggesting adaptive post-translational regulation of the pyruvate dehydrogenase complex in response to restored TPP availability rather than simple enzymatic activation or transcriptional remodeling. As elevated PDHA1 phosphorylation is normally associated with reduced PDC activity (43, 44), this may represent a counter-regulatory response to restored thiamine sufficiency. However, PDHA1 phosphorylation is a regulatory modification of the PDC and does not directly reflect enzyme activity or indicate alterations in other thiamine-dependent enzymatic systems. Notably, although both vitamers resulted in comparable whole-blood TPP levels at steady state, allithiamine-treated mice exhibited a significantly higher pPDHA1/PDHA1 ratio as early as 1 day after supplementation compared with thiamine-treated animals. This finding supports our suggestion that allithiamine may alter thiamine tissue distribution as earlier mentioned or intracellular availability, leading to a distinct temporal pattern of PDHA1 phosphorylation.

At the level of mitochondrial OXPHOS complex proteins, thiamine supplementation selectively increased hepatic ATP5A protein levels after 7 days, whereas allithiamine increased ATP5A levels after 1 day of supplementation. This observation may point to a limited adaptive response of mitochondrial complex V abundance to restored thiamine availability. However, the increase was moderate and restricted to ATP5A, a single representative subunit of complex V. Therefore, no direct conclusions can be drawn regarding complex V assembly, ATP synthase activity, or mitochondrial respiratory function. In addition, the absence of changes in representative markers of OXPHOS complexes I–IV argues against broader remodeling of the mitochondrial OXPHOS system under the present experimental conditions.

Of note, hepatic mRNA expression of genes encoding for thiamine transporters and thiamine pyrophosphate-forming enzymes remained unchanged compared with thiamine-depleted mice, indicating that the effects of thiamine and allithiamine do not involve transcriptional upregulation of thiamine-related genes.

Despite the strengths of our complementary model, several limitations should be considered when interpreting the results. Importantly, dosing in both models was designed to detect vitamer-specific effects under submaximal rather than saturating conditions. Within this framework, allithiamine and thiamine showed comparable bioactivity. Thiamine deficiency has been linked to metabolic disorders such as obesity and type 2 diabetes, where reduced thiamine status and altered glucose metabolism have been reported (45). Notably, a substantial proportion of individuals with obesity exhibit thiamine deficiency despite excess caloric intake (46), and thiamine supplementation has been explored as a supportive strategy to improve metabolic control (47). The potential advantages of allithiamine under impaired absorption or transporter function remain speculative and require validation in disease-specific models.

In addition, the murine study was limited to a short intervention period, a single dose level, and the use of male animals only, which restricts the assessment of steady-state physiology, dose–response relationships, and sex-specific effects. Moreover, whole-blood thiamine measurements do not fully reflect tissue-specific distribution or long-term metabolic adaptation. The model further does not capture absorption kinetics, tissue distribution, or enzyme activity, and the absence of pharmacokinetic and formal bioavailability analyses precludes definitive conclusions regarding the bioequivalence of thiamine and allithiamine. Future studies should include direct assessment of PDHA1 activity and acetyl-CoA levels to clarify functional consequences. Overall, incorporating these parameters would enhance physiological relevance and mechanistic resolution. The model may serve as a platform to investigate thiamine bioactivity in disease contexts and to evaluate other micronutrients.

## 5 Conclusion

In conclusion, this study demonstrates the suitability of *Drosophila* and mouse models for comparative thiamine research and establishes allithiamine as a biologically active thiamine vitamer *in vivo*. Restoration of thiamine status primarily engages post-translational metabolic regulation rather than transcriptional remodeling, with evidence for distinct early regulatory dynamics of allithiamine. Moreover, the structural properties of allithiamine may confer potential advantages in clinical contexts of impaired thiamine transport or malabsorption. Further studies are required to refine the model and to evaluate allithiamine bioactivity and translational relevance of allithiamine in human settings.

## Data Availability

The raw data supporting the conclusions of this article will be made available by the authors, without undue reservation.
